# Double Whammy: Abscopal Effect and Pseudoprogression in a Case of Non-small Cell Lung Carcinoma With Brain Metastases

**DOI:** 10.7759/cureus.59099

**Published:** 2024-04-26

**Authors:** Pranjal Rai, Abhishek Mahajan, Shreya Shukla, Ujjwal Agarwal

**Affiliations:** 1 Radiodiagnosis, Tata Memorial Hospital, Homi Bhabha National Institute (HBNI), Mumbai, IND; 2 Imaging Department, The Clatterbridge Cancer Centre National Health Service (NHS), Liverpool, GBR

**Keywords:** brain metastases, non-small cell lung adenocarcinoma, abscopal effect, stereostatic radiation therapy, tumor pseudoprogression

## Abstract

Abscopal effect and pseudoprogression are terms used in modern oncological imaging. Abscopal effect refers to the elicitation of tumor response away from the site of primary disease. Pseudoprogression is the increase in size or enhancement of the treated tumor or the appearance of new lesions that remain stable or show subsequent decrease without any change in therapy. Both of these are known to be associated with radiation therapy. We present a case of adenocarcinoma of the lung, which developed both these phenomena throughout the course of their therapy. Out-of-target responses secondary to radiotherapy have been discussed extensively in the literature and may pave the way for future oncological management as the targeted therapies become more specific. At the same time, atypical, however not uncommon, phenomena such as pseudoprogression should always be kept in the back of a clinician's mind as further course of clinical management may change.

## Introduction

The concept of utilizing localized radiation to elicit tumor responses away from the target is a phenomenon dating way back to the 1950s, which was originally described by Mole [1] using the term 'Abscopal effect'. The term comes from the Latin ab (meaning away) and Scopus (meaning target or mark). The term essentially describes the indirect anticancer effect secondary to the application of local ionizing radiation anywhere in the body. While it was originally described for only radiation therapy, strides have been made in the field of synergizing local radiotherapy and immunotherapy to produce the aforementioned out-of-target responses. Efforts have also been made in trying to understand this complex multi-layered phenomenon which was poorly understood at the start of this century. Pseudoprogression is a phenomenon that manifests on imaging as an initial increase in tumor size or enhancement, followed by subsequent stabilization or decrease, all occurring in the absence of modifications to the ongoing treatment regimen. As radiotherapy is a mainstay of management of multiple cancer treatments, around half the cancer patients [2], both abscopal effect and pseudoprogression have emerged as a curve ball for modern clinicians that can alter a patient's clinical management if the radiologist does not approach it with caution. Here, we present a patient with brain metastases in a case of non-small cell adenocarcinoma of the lung, which demonstrated the phenomenon of both abscopal effect and pseudoprogression.

## Case presentation

**Figure 1:**
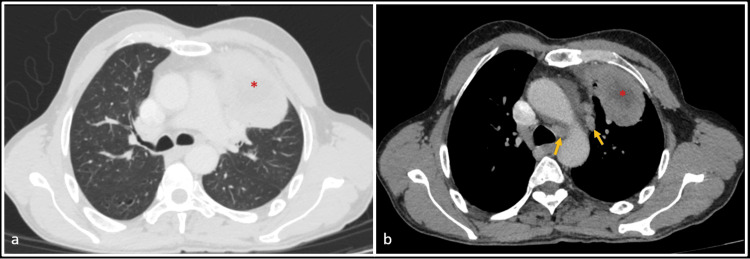
CT scan images of the patient's lungs (axial view) Axial contrast-enhanced computed tomography (CT) scans with lung (a) and soft tissue windows (b) show a large heterogeneously enhancing mass in the left upper lobe (red asterisk). Enlarged suspicious para-aortic and left lower paratracheal nodes are also seen (yellow arrow).

A 59-year-old female with a history of left upper lobe lung adenocarcinoma (Figure 1) had undergone a video-assisted left upper lobectomy with mediastinal lymph node dissection five years ago. Immunohistochemistry analysis of the primary tumor revealed Epidermal growth factor receptor (EGFR) mutation with Kirsten rat sarcoma (KRAS) codons 12 and 13 and Anaplastic lymphoma kinase (ALK) negative status. Two years post-operatively, she started experiencing neurological symptoms. Magnetic resonance imaging (MRI) of the brain demonstrated at least five enhancing neuroparenchymal enhancing lesions in the supratentorial and infratentorial compartments, consistent with brain metastases. The patient underwent Whole brain radiation therapy (WBRT) (30Gy for 10 fractions) for the metastases, followed by Afatinib for three years.

**Figure 2:**
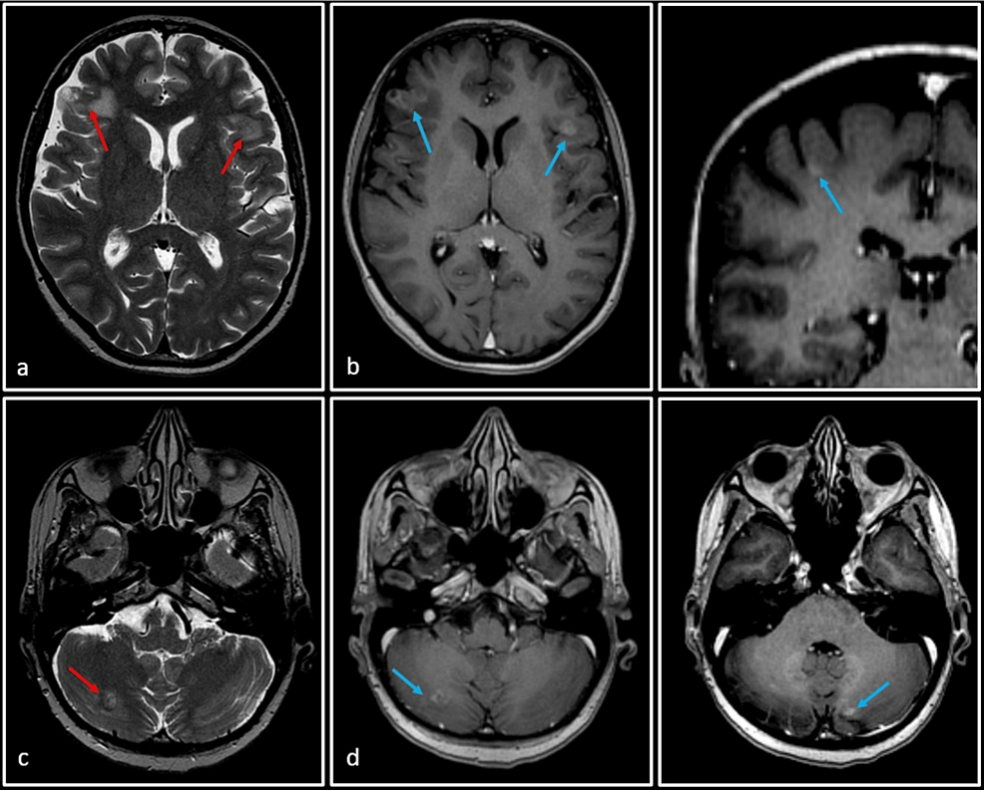
Follow-up MRI of the patient T2-weighted images (a and c) and post-Gadolinium T1-weighted images (b, d, e and f) show two ill-defined lesions in bilateral frontal lobes and one in the right cerebellar hemisphere (red arrows), with associated perilesional edema. The lesions are better appreciated on post-Gadolinium T1-weighted images (blue arrows), where a total of five lesions are seen: two in bilateral frontal lobes, two in bilateral cerebellar hemispheres, and the smallest one just inferior to the central sulcus on the right side at the grey-white matter junction. All these lesions were consistent with known metastases (post-whole brain radiation therapy).

The patient presented to our institution due to her recent clinical deterioration and worsening neurological symptoms. A follow-up MRI showed progression of brain lesions (Figure 2). A total of five lesions were seen: two in the bilateral frontal lobes, two in bilateral cerebellar hemispheres, and one just inferior to the right central sulcus at the grey-white matter junction. Due to their larger size, the first four lesions were selected to be treated with stereotactic radiosurgery (SRS). The fifth lesion, deemed too small for SRS at this time, would be monitored and considered for future treatment if necessary. Each cerebellar lesion received a single-fraction dose of 15.4 Gy. The right frontal lobe and left frontal lobe were treated with single-fraction doses of 18 Gy and 19 Gy, respectively. Additionally, osimertinib was initiated at a dose of 80 mg orally once daily.

**Figure 3:**
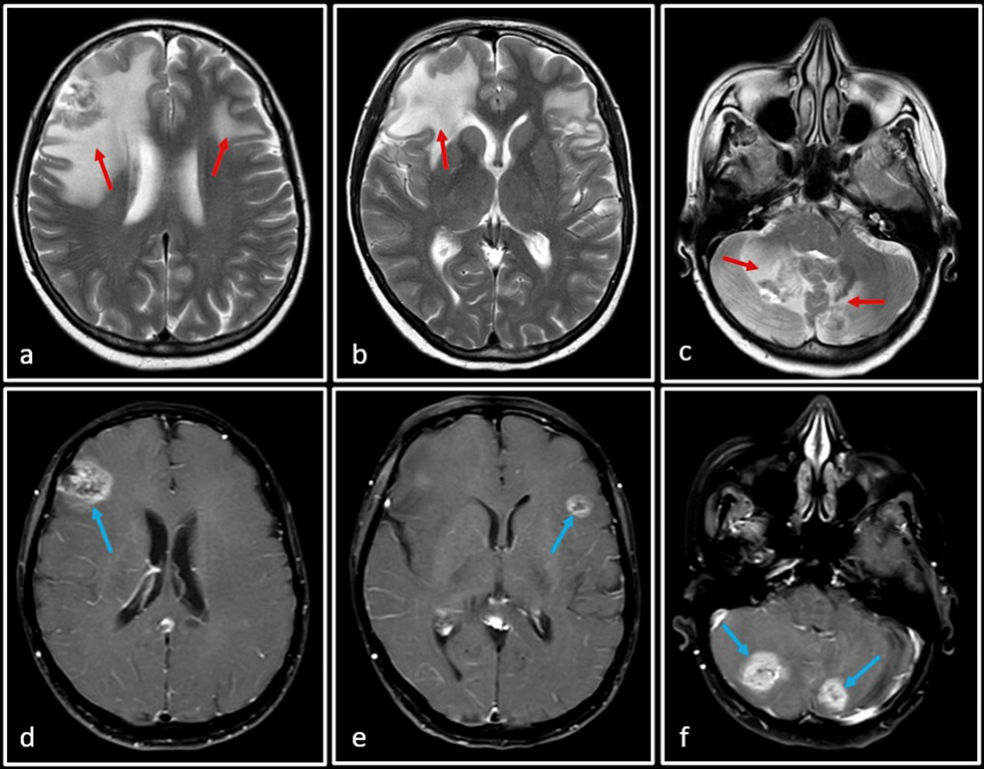
Follow-up MRI after three months of SRS to the frontal and cerebellar lobe lesions SRS: Stereotactic radiosurgery T2-weighted (a, b and c) and post-Gadolinium T1-weighted (d, e and f) images. There is an increase in size and enhancement of previously seen lesions (blue arrows) with an increase in the associated perilesional edema (red arrows). A working diagnosis of pseudoprogression was considered at this point of time and a follow-up MRI was requested.

Subsequent to SRS, a follow-up MRI performed at three months demonstrated a modest increase in the size of the bifrontal and cerebellar lesions. However, no new lesion was seen (Figure 3). Based on these findings, a working diagnosis of SRS-induced pseudoprogression was considered. This is supported by the typical presentation of pseudoprogression within this time frame, often resolving or stabilizing without requiring treatment modification. To confirm this diagnosis, a subsequent MRI was recommended at eight weeks. 

**Figure 4:**
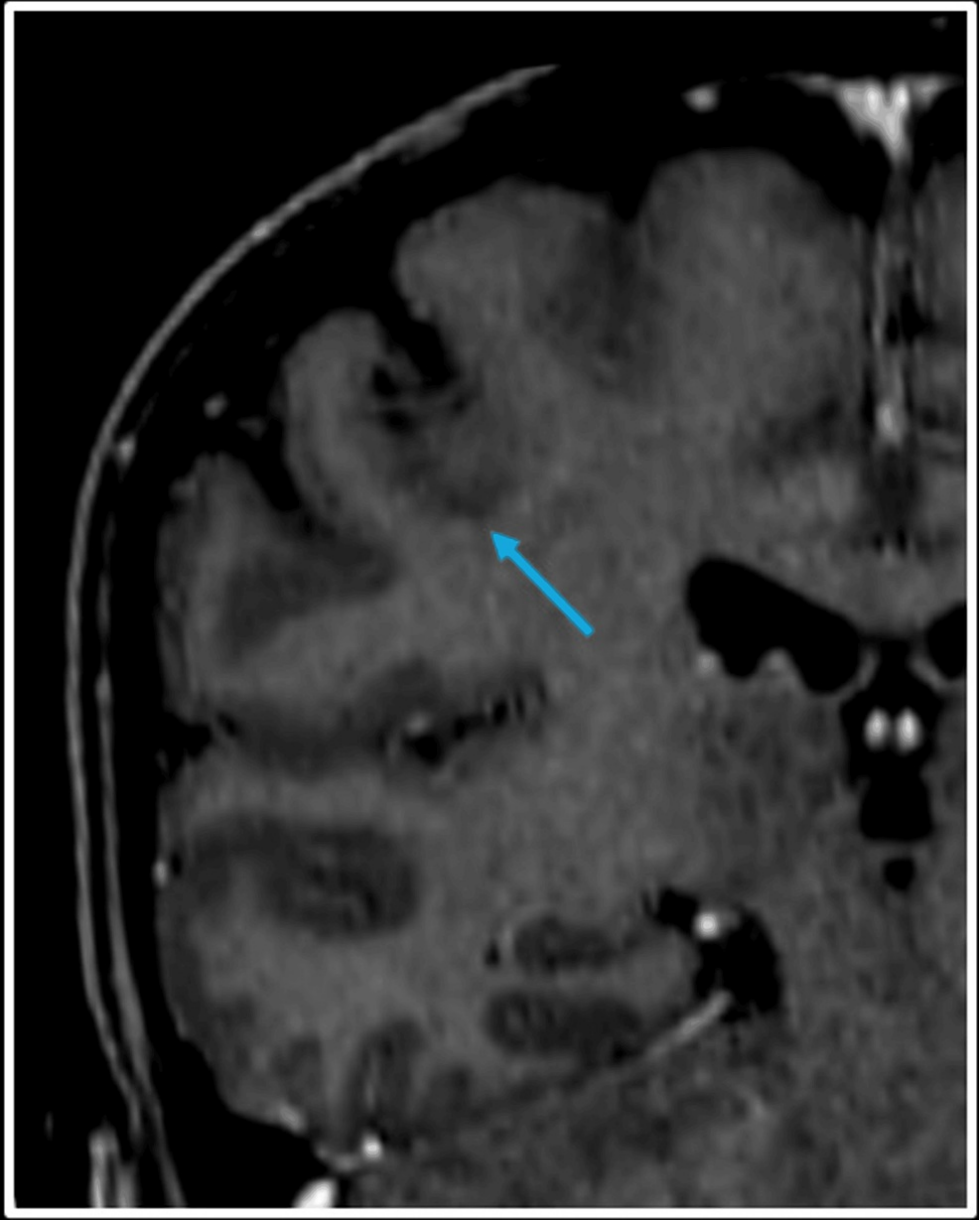
Coronal post-Gadolinium T1-weighted MRI The image shows the resolution of the previously seen non-irradiated lesion at the grey-white matter junction inferior to the right central sulcus (blue arrow).

In the same study, it was observed that the lesion just inferior to the central sulcus on the right side showed spontaneous resolution and was no longer appreciated in the MRI study (Figure 4). This observation raised the possibility of an abscopal effect.

**Figure 5:**
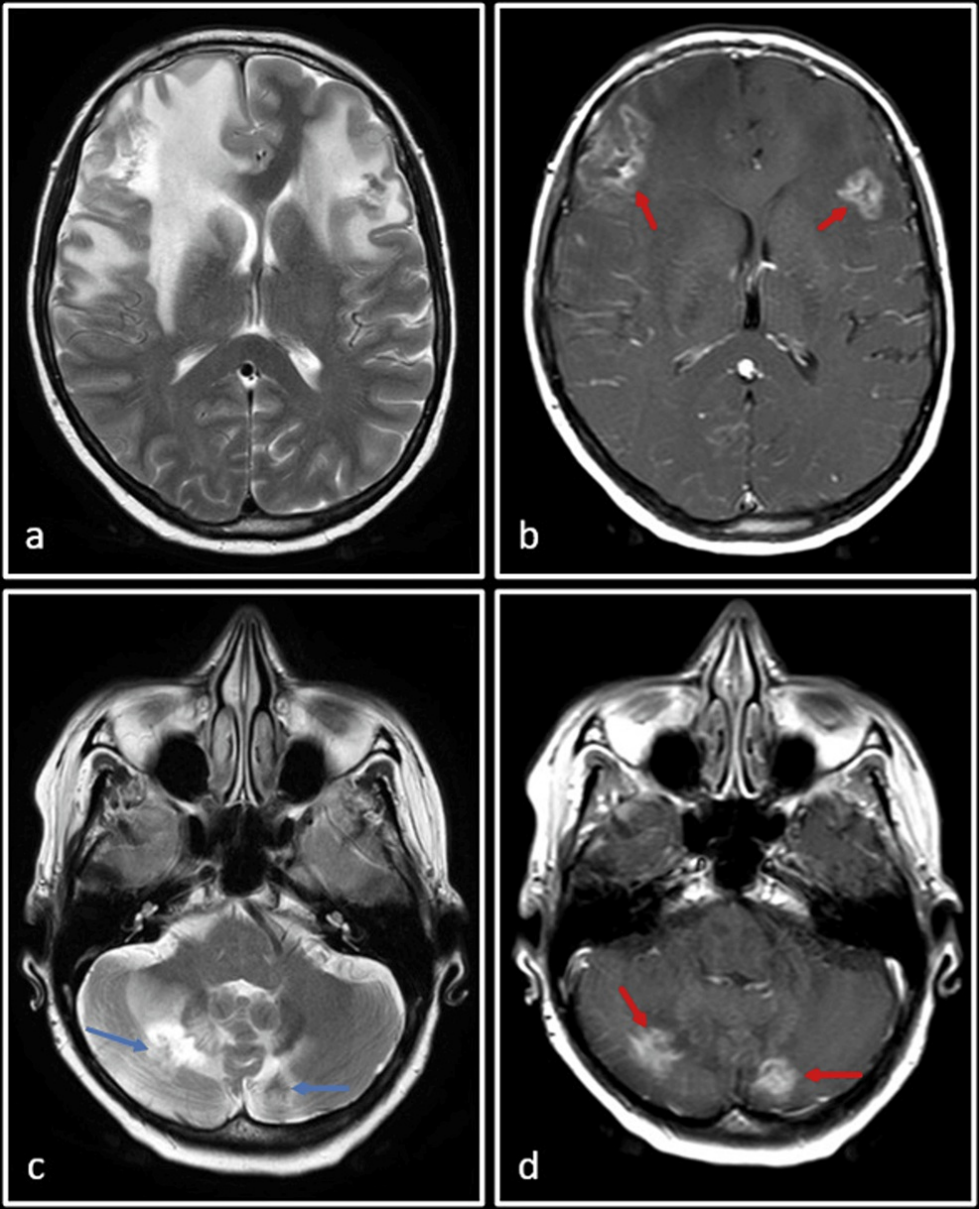
Follow-up MRI after six weeks T2-weighted (a and c) and post-Gadolinium T1-weighted (b and d) images. There is a decrease in size and enhancement in all the visualized lesions involving bilateral frontal (a and b) and cerebellar lobes (c and d). The lesions now show peripheral feathery enhancement (red arrows) which suggests post-treatment changes.

Subsequent follow-up with multifunctional MRI (perfusion imaging and spectroscopy) showed a marginal decrease in size and perilesional edema around all the irradiated lesions. Feathery "sieve-like" enhancement was also noted around these lesions without any significant hyperperfusion on arterial spin labeling (ASL) sequences. These changes were suggestive of post-treatment response within the lesions (Figure 5). Osimertinib was continued and the patient continued routine follow-ups.

**Figure 6:**
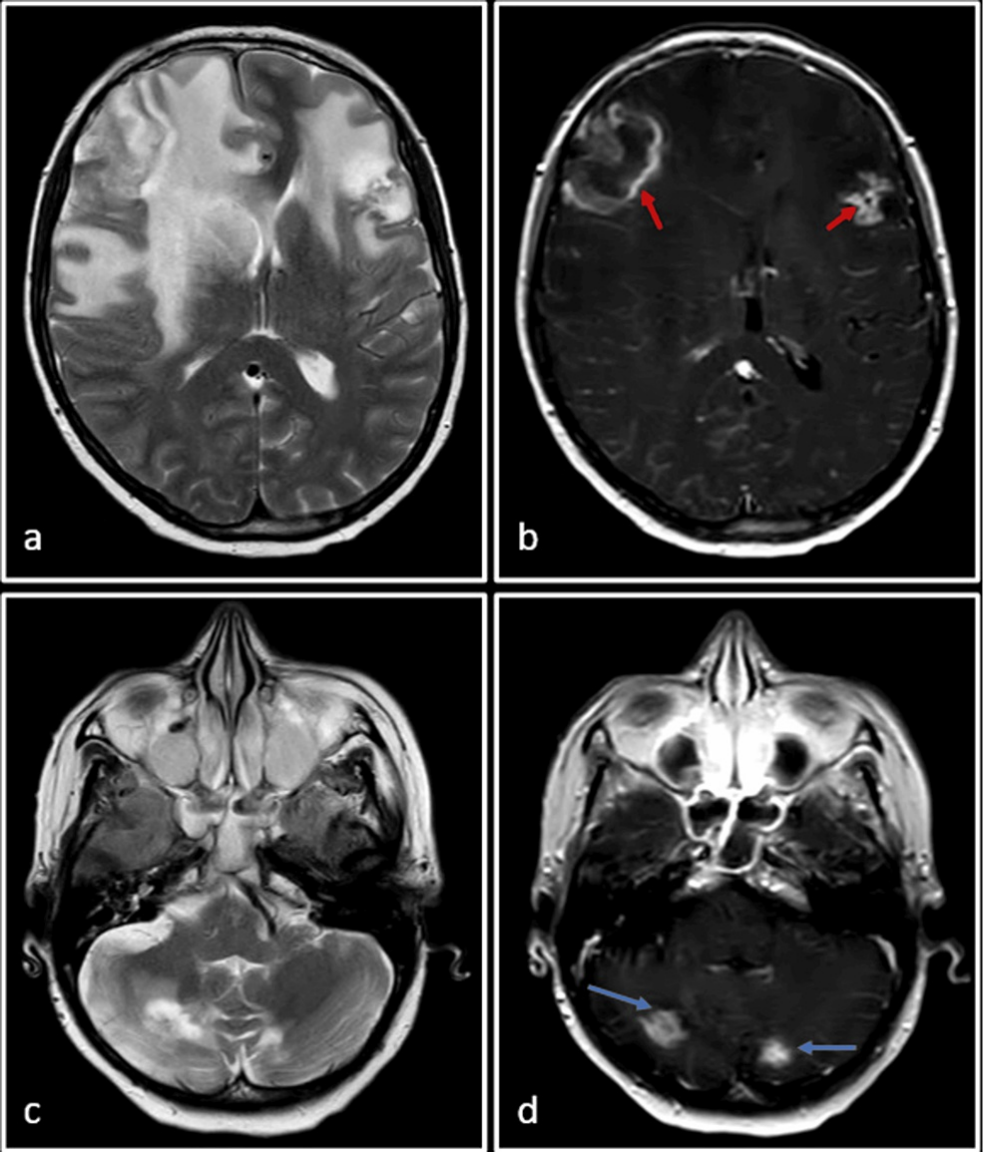
Follow-up MRI after 12 months T2-weighted (a and c) and post-Gadolinium T1-weighted (b and d) images. Note the increase in the size of the right frontal lobe lesion (a and b) with new onset peripheral nodular enhancement (red arrow) in both the frontal lobe lesions, consistent with true progression. The cerebellar lesions are unchanged in size (blue arrows).

Another six months later, the patient presented with headache, vomiting, increased frequency of micturition, and decreased cognition. MR brain with spectroscopy and perfusion was done, which showed an increase in the size of the right frontal lobe lesion with restricted diffusion, disproportionate perilesional edema, and increased mass effect (Figure 6). Excision of right frontal lobe lesion was done for palliation of symptoms and guarded prognosis was explained to the patient's relatives. Histopathology confirmed metastatic adenocarcinoma with genomic sequencing showing EGFR exon 19 deletion, Mesenchymal epithelial transition (MET) amplification, cyclin-dependent kinase -4 (CDK4) mutation, and murine double minute 2 (MDM2) mutation. The patient had symptomatic improvement post-surgery. A year later, the patient presented with drowsiness and decreased responsiveness and eventually succumbed.

## Discussion

Ionizing radiation therapy has been known to show out-of-target responses in the past due to the enhancement of tumor immunogenicity [3]. This has been termed the abscopal effect, and the exact pathology is still an enigma for many. A variety of underlying biological events can be hypothesized, the most important being the immune-mediated effect. This immune-mediated effect has been shown to hold some weight, as evidenced by various research models that have concluded or are underway. A proof-of-principle trial by Golden et al. [4] demonstrated abscopal responses in 27% of their patients by using fractionated local small-dose radiation along with GM-CSF (granulocyte-macrophage colony-stimulating factor) in addition to their previous standard systemic regimen. Those who developed such responses also had better overall survival.

The question as to why the abscopal effect doesn't occur more frequently in patients undergoing radiotherapy can be explained by the physiology behind it. The abscopal effect has essentially been summed up as antitumor immunity elicited secondary to radiotherapy and has been demonstrated in several cancer types, including lung, renal, hepatocellular, lymphoma, and melanomas [2]. In our case, it was demonstrated in metastatic non-small cell carcinoma of the lung.

Radiation acts as a physical aggressor and acts by the use of ionizing radiation, which either directly kills the cancer cells or causes genetic changes causing cancer cell death [2]. While radiation may play a key role in the abscopal effect, it may not be the only factor at play. Radiation treatments can be either immunosuppressive or immunostimulatory depending on the tissue, tumor context, and the host's immune response [5,6]. A permissive host environment, along with the correct timing and prerequisite immunomodulatory signals, may all be necessary to trigger the abscopal response.

Danger signals and inflammatory cytokines produced by cell death after radiation-induced cell injury promote the ability of dendritic cells to cross-present released antigens to the T-cells [7]. This recruitment of the immune system converts the tumor essentially into an "in-situ personalized vaccine". The immune system can subsequently reject the irradiated tumor as well as systemic metastases associated with the tumor. This forms the basis of the abscopal effect. Studies have shown that the radiation regimen (fractionated or single dose), site of radiation, and tumor histologic characteristics may all contribute to the effect [8]. In our case, four of the metastatic lesions (in both frontal lobes and cerebellar hemispheres) received SRS, with the right and left frontal lobes receiving 18 Gy and 19 Gy, respectively, and the cerebellar lesions receiving 15.4 Gy each in a single fraction. However, no immunotherapy was administered to the patient throughout the course of her treatment.

Another phenomenon that was demonstrated in our case was pseudoprogression of brain metastases. Pseudoprogression is a form of an unconventional response pattern seen in patients with gliomas being treated with chemoradiotherapy and essentially in most tumors being treated with immunotherapy [9]. It is characterized by the appearance of new lesions or growth in tumor size, subsequently followed by a decrease in tumor burden. These changes are confirmed by biopsy or radiological imaging [9]. Additional use of biomarkers such as circulating tumor deoxyribonucleic acid (ct-DNA), which is the DNA fragments from dying tumor cells that are freely circulating in the bloodstream, can also be used to indirectly assess tumor burden in patients with suspected pseudoprogression [10].

Imaging features suggestive of pseudoprogression include the new or enlarging areas of contrast enhancement occurring early after radiotherapy (usually within three to four months), which usually stabilizes or reduces on subsequent imaging without any change in therapy. In comparison, radiation necrosis which usually occurs six months to several years after radiation therapy, is characterized by the absence of any intermediate areas on T2-weighted images, hyperintense areas on Fluid-attenuated inversion recovery (FLAIR) sequences, new onset areas of diffusion restriction or with new onset feathery enhancement in the areas that have been covered in the radiation field [11]. MRI perfusion may play a key role in differentiating true progression from pseudoprogression. It has been observed in existing literature that pseudoprogression lesions are typically characterized by lower perfusion and higher permeability. Thus, a characteristic decrease in relative peak height and relative cerebral blood volume is associated with an increase in percent signal recovery (PSR), which is associated with pseudoprogression. Similarly, MR Spectroscopy is of value in dubious cases, where elevated lipid signal and absence of choline or low choline/NAA (N-Acetylaspartic acid) ratio help rule out true progression and generally favor pseudoprogression [9].

SRS-induced pseudoprogression was reported after a median interval of seven to 11 months, and in our case, was seven months [11]. The surgical decision was taken only after the patient became symptomatic two years later.

The key teaching point from our case is that timely and accurate diagnosis of pseudoprogression by the radiologist is important as appropriate clinical decisions and second-line drug therapy may be added or modified if it is erroneously reported as true progression. Thus, the knowledge of imaging findings is quintessential, and advanced MRI techniques may be used in equivocal cases. Secondarily, the knowledge of the abscopal effect should also be known to both the radiologist and the clinician so that therapeutic strategies such as immunotherapeutic agents can be implemented and modified to elicit the best possible treatment response in cancer patients.

## Conclusions

This case highlights two important considerations in managing brain metastases from lung cancer, which are the abscopal effect and pseudoprogression. The former aims to remind us that radiation therapy, with or without immunotherapy, when incorporated into cancer management, holds the promise of complementing currently available modalities such as chemotherapy, hormonal therapy, and biologic agents for the management of disseminated malignancies as it harnesses the immune system to target non-irradiated lesions. Secondly, identifying pseudoprogression is important for radiologists as subsequent management can change drastically. Hence, the need for the establishment of monitoring and evaluation criteria is of paramount importance. Further studies need to be done to elucidate its exact mechanism.
